# Abcès cérébral compliquant une cardiopathie congénitale: à propos de 7 cas à l'institut de cardiologie d'Abidjan

**DOI:** 10.11604/pamj.2015.22.109.6910

**Published:** 2015-10-08

**Authors:** Yves N'da Kouakou N'goran, Micesse Tano, Fatoumata Traore, Inès Angoran, N'guetta Roland, Aké Evelyne Traboulsi, Konan Serge Yao, Kouadio Euloge Kramoh, Maurice Guikahue Kakou

**Affiliations:** 1 Institut de Cardiologie d'Abidjan, Côte d'Ivoire; 2 Service de Neurochirurgie CHU de Yopougon, Côte d'Ivoire

**Keywords:** Abcès cérébral, cardiopathie congénitale, tétralogie de Fallot, cerebral abscess, congenital heart disease, tetralogy of Fallot

## Abstract

Le diagnostic précoce des cardiopathies congénitales (C.C) a une incidence positive sur leur évolution. En effet diagnostiquées tard ou non traitées elles peuvent se compliquer. L'abcès cérébral est une complication des C.C cyanogènes qui est rare dans les pays développés. Notre objectif était d'analyser à travers une revue bibliographique les particularités de 7 cas de C.C compliquées d'abcès cérébral découvertes et prises en charge dans un service de cardiologie pédiatrique de l'Institut de Cardiologie d'Abidjan. La tétralogie de Fallot était la cardiopathie congénitale la plus fréquente. Le traitement a été médical et/ou chirurgical. Seule la réalisation de la cure complète des cardiopathies congénitales peut permettre la prévention de l'abcès cérébral.

## Introduction

Le diagnostic précoce des Cardiopathies Congénitales (C.C) a une incidence positive sur leur évolution. En effet diagnostiquées tard ou non traitées elles peuvent se compliquer. Selon le type de cardiopathie, il peut s'agir: d'une insuffisance cardiaque, d'endocardite infectieuse, d'une polyglobulie sévère, d'un accident cérébro-vasculaire ou d'un abcès cérébral. L'abcès cérébral en particulier est une collection de pus dans le parenchyme cérébral. C'est une complication classique des C.C cyanogènes, décrite depuis 1814 [1]. Il survient plus tardivement, au-delà de l’âge de 2 ans. L'abcès cérébral devrait donc devenir rare, puisque les C.C sont généralement opérées avant cet âge notamment dans les pays développés [2]. En Afrique, les publications sur l'abcès cérébral et C.C sont rares. Nous n'avons pas retrouvé de publications concernant l'abcès cérébral et C.C en Côte d'Ivoire. Quelle est la situation dans notre région où non seulement les diagnostics sont tardifs, mais la prise en charge chirurgicale est tardive ou inexistante? L'objectif de notre étude est de Décrire les caractéristiques épidémiologiques, les données cliniques, para-cliniques, la prise en charge et l’évolution de cette complication.

## Méthodes

Il s'agissait d'une étude prospective réalisée du 01/02/2005 au 31/12/2013. Elle a concerné les patients, atteints d'abcès cérébral compliquant une C.C, hospitalisés dans le service de Cardiologie pédiatrique de l'Institut de Cardiologie d’ Abidjan. Pour chaque patient, ont été analysées les données épidémiologiques, cliniques, para cliniques, thérapeutiques et évolutives. L'analyse statistique a été faite à l'aide des logiciels Epi Data et Excel.

## Résultats

**Figure 1:**
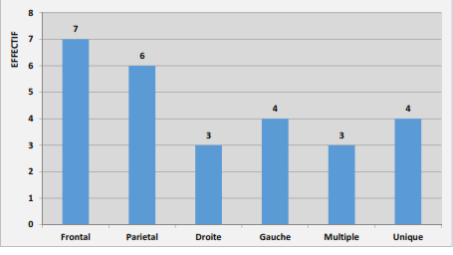
Répartition des patients en fonction de la situation et du nombre de l'abcès cérébral

Nous avons colligé 07 dossiers d'abcès cérébral compliquant une C.C sur 1231 patients hospitalisés durant la période d’étude, la prévalence hospitalière a donc été de 0.6%. Dans notre population d’étude 3 (42,86%) patients étaient de sexe masculin et 4 (57.14%) de sexe féminin. Le sexe ratio était de 0.75. L’âge moyen était de 12.6 ± 11.7 ans avec des extrêmes de 2 ans à 33 ans. La répartition des patients en fonction des classes d’âge apparaît dans le Tableau 1. Les signes se rapportant à la C.C se retrouvaient dans tous les cas. Les signes de l'abcès cérébral étaient: la fièvre, les céphalées, les vomissements, la convulsion, le trouble de la conscience, le déficit neurologique et les signes d'irritation méningée. La fièvre était retrouvée chez tous les patients. Les céphalées ont été retrouvées dans 5 cas, il s'agissait de patients ayant un âge supérieur à 2 ans. Les vomissements étaient retrouvés dans 4 cas, vomissements faciles en jet et postprandiaux. Les convulsions ont été observées dans 6 cas. Elles étaient partielles dans un cas. Un trouble de la conscience était objectivé chez 03 patients avec un score de Glasgow calculé entre 8 et 10 /15. Les déficits neurologiques retrouvés dans 03 cas étaient à type d'hémiplégie associée à une paralysie faciale dans un cas. Trois patients présentaient un syndrome méningé. Dans notre étude 05 patients étaient porteurs d'une tétralogie de Fallot soit 71,4% (dont un avait bénéficié d'un traitement palliatif: intervention de Blalock), 1 patient porteur d'un ventricule unique avec dextrocardie et situs inversus (14,3%) et un autre une oreillette unique (14,3%). Les tétralogies de Fallot avaient en moyenne un bon indice de Nakata à 267.4 avec des extrêmes allant de 230 à 338. Il n'y avait pas de végétations retrouvé à l’échographie cardiaque. Le siège de l'abcès cérébral (Figure 1) était à la fois frontal et pariétal dans 06 cas et dans 01 cas il était frontal. Dans 03 cas il intéressait la partie droite du cerveau et dans 04 cas la partie gauche. L'abcès cérébral était unique dans 04 cas et multiple dans 03 cas. La numération globulaire avait objectivé une polyglobulie dans tous les cas: un taux d'hémoglobine moyen 19.7g/dl extrêmes allant 15 à 23 g/d; un taux d'hématocrite moyen à 60% avec des extrêmes allant de 58 à 65%; le nombre de globule rouge moyen était de 8,4.106 élt/mm3 avec des extrêmes allant de 5 à 10.10^6^ élts/mm^3^. Une hyperleucocytose à prédominance neutrophile a été également objectivée chez tous les patients. La saturation artérielle en oxygène moyenne était de 87.1% avec des extrêmes allant de 70 à 95%. Il n'y avait pas eu d'isolement de germes dans le liquide purulent après drainage. La sérologie rétrovirale était négative chez tous les patients. Le traitement médical a été réalisé dans tous les cas, qui a consisté à une tri antibiothérapie pendant trente-six jours en moyenne (cinq semaines un jour). Les molécules utilisées étaient une céphalosporine de 3^ème^ génération ou l'amoxicilline+ acide clavulanique 100 à 200 mg/kg/jour, un aminoside 5mg/kg/jour et un métronidazole 30mg/ kg/ jour. Tous les patients ont reçu une antibiothérapie à large spectre avant la confirmation de l'abcès cérébral. Le traitement antiépileptique tel que l'acide valproïque (dépakine^®^) a été donné pendant au moins un an à tous nos patients. Le traitement chirurgical consistait à une trépano-ponction avec drainage dans le service de neurochirurgie du CHU de Yopougon. L’évolution a été favorable pour tous les patients après le traitement de l'abcès cérébral. Il n'y a pas eu de séquelles neurologiques ni de décès: La réalisation de l'EEG après trois ans chez trois patients était normale, après deux ans chez deux patients était normale et un an après chez un patient était normale. Après le traitement de l'abcès cérébral, deux patients ont été opérés (un palliatif et une cure complète); deux en attente d'une cure complète, un décédé de malaise anoxique, un perdu de vue et un suivi en consultation de cardiologie.

**Tableau 1:** Répartition des patients selon les classes d’âge

Tranche d’âge (an)	Effectif	(%)
**< 5**	2	28,6
**[5, 10 [**	2	28,6
**[10, 15 [**	1	14,2
**> 15**	2	28,6
**TOTAL**	7	100

## Discussion

Durant notre période d’étude de 8 ans, nous avons observé une prévalence hospitalière de 0.6% d'abcès cérébraux compliquant les C.C. Frazier et al. [3] note une prévalence d'abcès cérébraux avec une C.C entre 6 et 51%. Ils ont des prévalences aussi élevées parce qu'ils avaient comme population d’étude les patients atteints d'abcès du cerveau. Contrairement à la nôtre où la population d’étude était des patients atteint de C.C. Ils avaient effectué l’étude dans un service de neurochirurgie et non de cardiologie pédiatrique. Mais en ce qui concerne le nombre de cas dépisté il est superposable à celui de ces auteurs (variant entre 7 et 20 cas) pour une durée d’étude semblable (Tableau 2). L'abcès cérébral complique rarement les C.C avant l’âge de 2 ans et augmente continuellement jusqu’à l’âge de 12 ans. Le risque instantané à cet âge est estimé à 1,75 ± 0,12%, mais décroît après [4]. Le pic de fréquence se situe entre 4 ans et 7 ans selon Lumbiganon et al [5]. Dans notre étude aucun patient n'avait un âge inférieur à 2 ans. L’âge moyen était de 12.6 ± 11.7 ans qui est supérieur à celui d'Atiq [4] 5.6± 4.4 ans et Thiam [6] 9.6 ans. Cette différence était due au fait que dans notre série, il y avait deux patients adultes dont l’âge était supérieur à 15 ans (24 et 33 ans), seulement un patient était âgé de 2 ans. Dans la littérature le tableau clinique est dominé par la tétrade: céphalée, vomissement, fièvre, convulsion [7]. Les signes cliniques les plus fréquemment rencontrés dans notre série étaient la fièvre (chez tous nos patients), les convulsions (85.8%), les céphalées (71.4%) et les vomissements (57.1%). La prédominance de la fièvre est retrouvée par des auteurs comme Kai-Liang et al. [8] qui cite l'analogie avec d'autres auteurs. En ce qui concerne les troubles neurologiques, on a observé un déficit neurologique, un trouble de la conscience et des signes d'irritation méningée dans 43% chacun. Ces signes ont été retrouvés dans la littérature [7], associés à un oedème papillaire. Dans notre étude l’œdème papillaire n'a pas été retrouvé parce qu'il n y a pas eu de réalisation de fond d’œil. La C.C sous-jacente (Tableau 3) retrouvée dans notre étude était la tétralogie de Fallot dans 5 cas, le ventricule unique dans un cas, l'oreillette unique dans un cas. Ce sont toutes des C.C cyanogènes. Elles constituent un facteur important prédisposant à la constitution d'un abcès du cerveau, ce qui représente 25 à 46% des cas selon Atiq et al. [4].

**Tableau 2:** Abcès du cerveau sur cardiopathie congénitale selon les différentes études

Auteurs	Abcès Sur CC	Durée de l’étude
**Notre serie (2014)**	7	08 ans
**Thiam** ^**6**^ **(2010)**	10	08 ans
**Kai-Liang** ^**8**^ **(2008)**	7	11 ans
**Atiq** ^**4**^ **(2006)**	11	10 ans
**Janson** ^**5**^ **(2004)**	9	10 ans
**Abdullah** ^**5**^ **(2001)**	20	07 ans
**Ciuera** ^**7**^ **(1999)**	20	08 ans
**Guérin** ^**5**^ **(1997)**	7	15 ans

**Tableau 3:** Répartition des patients selon la cardiopathie congénitale

Cardiopathie congénitale	Effectif	(%)
Tétralogie de Fallot	5	71,4
Ventricule unique + situs inversus	1	14,3
Oreillette unique	1	14,3
**Total**	07	100

La tétralogie de Fallot est La C.C cyanogène la plus concernée dans notre étude. Elle est responsable d'un shunt intracardiaque droite-gauche, le sang n'est plus filtré à travers la circulation pulmonaire [6]. Ainsi les bactéries échappent à leur phagocytose dans les poumons. Une étude [4] a révélé une réduction des fonctions phagocytaires et bactéricides des leucocytes chez les enfants atteints de C.C cyanogène dont la plus fréquente demeure la tétralogie de Fallot [5]. Nous n'avons pas retrouvé de cardiopathies non cyanogènes mais dans la littérature il est rapporté des cas abcès cérébraux associés à une communication inter ventriculaire ou une communication inter auriculaire [7]. Les abcès étaient situés dans les régions frontale et pariétale du cerveau dans notre étude. Ces localisations sont les plus fréquentes [4]. Les abcès peuvent être multiples ou uniques, les abcès multiples sont fréquents chez les patients immunodéprimés avec endocardites infectieuse [4]. Dans notre étude il n'y avait pas de cas d'immunodépression ni d'endocardite infectieuse mais on avait des abcès multiples (3cas/7). Le germe le plus fréquemment isolé, dans l'abcès du cerveau compliquant une C.C cyanogène est le streptococcus milleri [4]. Dans notre étude, aucun germe n'a été retrouvé après culture du liquide de drainage. Cela peut être dû à des conditions de prélèvements en milieu aérobie (détruisant les anaérobies). Dans la littérature on trouve un taux élevé de culture stérile [7]. Cette élévation étant due au non-respect des procédures de prélèvement bactériologique et à l'utilisation d'une antibiothérapie à large spectre [7]. La numération globulaire avait objectivé une polyglobulie dans tous les cas. Elle a été décrite comme le facteur de risque le plus important, dans le développement de l'abcès du cerveau, chez les patients porteurs de C.C cyanogène [4].

Le traitement médical dans notre pratique a associé une tri antibiothérapie, les molécules utilisées étaient une céphalosporine de 3^ème^ génération ou l'amoxicilline + acide clavulanique 100 à 200 mg/kg/jour, une aminoside 5mg/kg/jour et une métronidazole 30mg/ kg/ jour. A défaut de trouver un germe nous avons utilisé une antibiothérapie à large spectre c'est-à-dire agissant à la fois sur les germes à gram positif, gram négatif et les anaérobies. Dans la littérature il est recommandé en cas d'abcès du cerveau et C.C cyanogène d'utiliser l'association chloramphénicol et aminopenicilline en première intention [4]. Le traitement chirurgical a consisté à faire une trépano-ponction avec drainage. C'est une technique relativement simple, efficace et bien tolérée chez ces patients en mauvaise condition du fait d'une basse saturation en oxygène, donc incapables de supporter une longue et lourde chirurgie du genre exérèse [6]. La trépano-ponction est la seule technique appliquée dans notre pays. D'autres techniques existent. L'excision par une craniotomie peut être efficace. Cependant il n'est pas conseillé pour les abcès profonds ou localisés dans les zones éloquentes. Les tentatives d'excision peuvent entraîner une morbidité neurologique selon Ciuera et al. [7]. L'aspiration stéréotaxique est déjà le traitement standard accepté partout dans les pays développés [2]. Le drainage endoscopique des abcès cérébraux est une autre modalité thérapeutique décrite dans la littérature [5]. Cette technique permet d'inspecter et de visualiser directement l'aspiration de la collection purulente, à la différence de l'aspiration stéréotaxique au cours de laquelle il y a un manque de contrôle visuel direct. En outre, les abcès multi loculés pourraient éventuellement être traités avec la technique endoscopique [9]. L’évolution a été favorable dans notre étude pour tous les patients, après le traitement de l'abcès cérébral. Il n'y a pas eu de séquelles neurologiques. Le seul cas de décès a été constaté 2 mois après l'abcès du cerveau, dans un contexte de malaise anoxique. Les séquelles neurologiques rapportées dans la littérature sont [7]: les crises épileptiques, le déficit neurologique, les troubles cognitifs. Dans notre étude nous avons donné des antiépileptiques (acide valproïque sel de sodium (dépakine^®^)) pendant un an au moins pour prévenir les crises épileptiques. La réalisation de l'EEG après trois ans chez trois patients était normale, après deux ans chez deux patients était normale et un an après chez un patient était normale. Dans la littérature, Jusqu′aux années 1970, le taux de mortalité était supérieur à 36%, mais elle a ensuite diminué après les années 1980 [10]. Pour la plupart des auteurs, le pronostic des abcès cérébraux sur cardiopathie cyanogène est mauvais avec une mortalité entre 27,5 et 71% [6]. Les facteurs de mauvais pronostic sont les abcès multiples, l'hypertension intracrânienne associée à une méningite et/ou une ventriculite. Il ne note aucune corrélation entre la mortalité et l’âge, le sexe, le type de germe, le site de localisation de l'abcès et le type de cardiopathie. Notre faible taux de mortalité peut s′expliquer par le diagnostic plus précoce résultant du développement de nouvelles modalités d′imagerie et de la rapidité de la prise en charge médicale et chirurgicale de ces abcès cérébraux.

## Conclusion

L'abcès cérébral compliquant une cardiopathie congénitale est rare en Côte d'Ivoire avant l’âge de deux ans. L’âge moyen de survenue est élevé. Les cardiopathies cyanogènes sont les plus concernées, essentiellement la tétralogie de Fallot. Le tableau clinique est dominé par des convulsions fébriles, céphalées et les vomissements. Le diagnostic est confirmé par le scanner crânio encéphalique. Le facteur de risque le plus important dans le développement de l'abcès est la polyglobulie. Le traitement est médical (antibiothérapie) et / ou chirurgical (par ponction drainage). La prévention nécessite la réalisation de la cure complète des cardiopathies congénitales.
